# Spatio-Temporal Dynamics of Caddisflies in Streams of Southern Western Ghats

**DOI:** 10.1673/031.010.4601

**Published:** 2010-05-12

**Authors:** S. Dinakaran, S. Anbalagan

**Affiliations:** Centre for Research in Aquatic Entomology, Department of Zoology, The Madura College (Autonomous), Madurai -625 011, Tamil Nadu, India

**Keywords:** diversity, habitat, seasonality

## Abstract

The dynamics of physico-chemical factors and their effects on caddisfly communities were examined in 29 streams of southern Western Ghats. Monthly samples were collected from the Thadaganachiamman stream of Sirumalai Hills, Tamil Nadu from May 2006 to April 2007. Southwest and northeast monsoons favored the existence of caddisfly population in streams. A total of 20 caddisfly taxa were collected from 29 streams of southern Western Ghats. *Hydropsyche* (Trichoptera: Hydropsychidae) were more widely distributed throughout sampling sites than were the other taxa. Canonical correspondence analysis showed that elevation was a major variable and pH, stream order, and stream substrates were minor variables affecting taxa richness. These results suggested that habitat heterogeneity and seasonal changes were stronger predictors of caddisfly assemblages than large-scale patterns in landscape diversity.

## Introduction

The Western Ghats has many streams and large rivers and is one of the biodiversity hotspots for terrestrial and freshwater organisms (Meyers et al. 2000; Dudgeon 1999). The biota of Western Ghats streams has been little studied, except for some groups of aquatic insects such as mayflies (Sivaramakrishnan et al. 1996) and dragonflies (Subramanian and Sivaramakrishnan 2002). There are few investigations of environmental influences on aquatic macroinvertebrate distribution in tropical areas, and most of them are quite recent. They include the influence of seasonal variation in a headwater stream (Julka et al. 1999; Anbalagan et al. 2004), temporal variation of functional feeding groups (Anbalagan 2005); habitat and microhabitat distribution patterns (Subramanian and Sivaramakrishnan 2005), and effects of land use (Subramanian et al. 2005). The Trichoptera are not a well-studied group in streams of Western Ghats (Dudgeon 1999). Studies on Trichoptera started only after the middle of the 19th century, and later study mainly focused on taxonomical, rather than ecological, aspects of this group (Dudgeon 1999). Only one study concerned ecological aspects (Dinakaran 2004). No studies have been performed in the Western Ghats that examined factors affecting large scale caddisfly distribution. In contrast, several examples have been done in Europe (Leuven et al. 1987; Wiberg-Larsen et al. 2000), North America (Ross 1963), and South Africa (de Moor 1992). The objective of the present study was to examine spatial and temporal dynamics of caddisfly communities in 29 streams of southern Western Ghats.

## Materials and Methods

### Sampling sites

**Figure 1.**
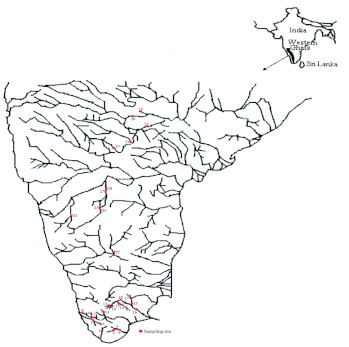
Sampling sites in the streams of southern Western Ghats. High quality figures are available online.

All totaled, 29 streams were surveyed across the three states (Kerala, Tamil Nadu and Karntaka) of southern Western Ghats. The mountains intercept the rain-bearing southwest monsoon winds and are consequently an area of high rainfall. The important east flowing rivers of southern Western Ghats are the Tampiraparani (east), Vaigai, Cauvery, Tungabhadra, Bhima and Krishna rivers. The Kallidai, Tampiraparani (west) and Kallar rivers are west-flowing. The present study was carried out in streams of seven river basins of southern Western Ghats, namely the Tampiraparani (Honey Falls, Shenbagadevi Falls, Five Falls and Chinna Kuttalam), Kallidai (Palaruvi streams 1–4 and the Kallidai river), Vamanapuram (Kallar), Vaigai (Thadaganachiamman Stream, Ayyanar Falls, Kumbakkarai Falls, Pampar Cascade downstream, Thalayar, Silver Cascade downstream, Kurusedi and Moolayaru), Tungabhadra (Thanikode, Boklapura halla, Sringeri Stream and Theerthahalli), Bhima (Sanjitha nathi, Kalgi and Kakini river) and Krishna river basins (Bonal, Upper Krishna and Kadarattar) (Figure 1). Monthly samples were collected from the Thadaganachiamman stream (Vaigai river basin) of Sirumalai Hills, Tamil Nadu from May 2006 to April 2007. Sampling sites included a variety of river types and reaches and riparian communities with and without structured vegetation.

### Sampling procedure

Air and water temperatures were recorded in the field. Dissolved oxygen, total dissolved solids, conductivity and pH were measured using a water analysis kit (Naina Solaris Limited, www.indianindustry.com). Latitude, longitude, elevation and basin location were determined by global positioning, GPS. Substrates were classified (Jowett et al. 1991) using the following criteria: <0.5 mm for mud/silt, 0.5–2 mm for sand, 2–64 mm for gravel, 65–256 mm for cobbles, and >256 mm for boulders. For statistical analyses, substrate composition was converted to a substrate index (Suren 1996):

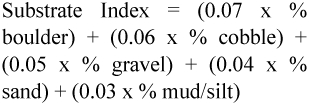

The average stream width and depth were calculated from three measurements with a calibrated stick from one transect across the channel. Current velocity of the stream was obtained by a flow meter. Canopy cover was measured using a densitometer, and dominant species of riparian vegetation were recorded for each sampling site.

For each study site and sampling occasion, three 50 × 50 cm benthic samples were taken at random locations from each riffle and pool. In riffle samples, caddisflies larvae were collected using 180-µm-mesh kick-nets, and 500-µm-mesh dip nets were used for pool sampling. Soon after collection, the specimens were preserved in 70% ethanol. All caddisfly larvae were identified to the lowest possible taxonomic level using available keys (Dudgeon 1999).

### Data analysis

Observations of the physical and chemical characters were recorded for each site on each sampling date, and the richness and density of the Trichoptera taxa were summarized as mean values, standard deviation and coefficient variation. Dissolved oxygen, conductivity and temperature were graphically presented to illustrate the seasonal changes in water quality. In each sampling station, diversity indices were estimated. Alpha diversity indices of the Shannon-Wiener diversity index and the Simpson diversity index, species richness index of Margalef, and evenness of index Pielou were calculated according to Ludwig and Reynolds (1988). Similarities in taxonomic composition were quantified using Jaccard's index (Sneath and Sokal 1973; Magurran 1988) based on a presence-absence matrix for the insect fauna of each stream. More specifically, similarity (S_ij_) between any pair of sites i and j is given by

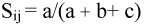

where a is the number of taxa shared in common, b is the number of taxa in site i but not site j, and c is the number of taxa in site j but not site i. Two-way Bray-Curtis analysis was performed using the results of Jaccard's index. Canonical correspondence analysis (CCA) was calculated, measuring the relationship between 11 environmental variables, taxa richness of caddisflies, and seasonal changes on caddisfly taxa among sampling sites (Legendre and Legendre 1998).

**Table 1.**
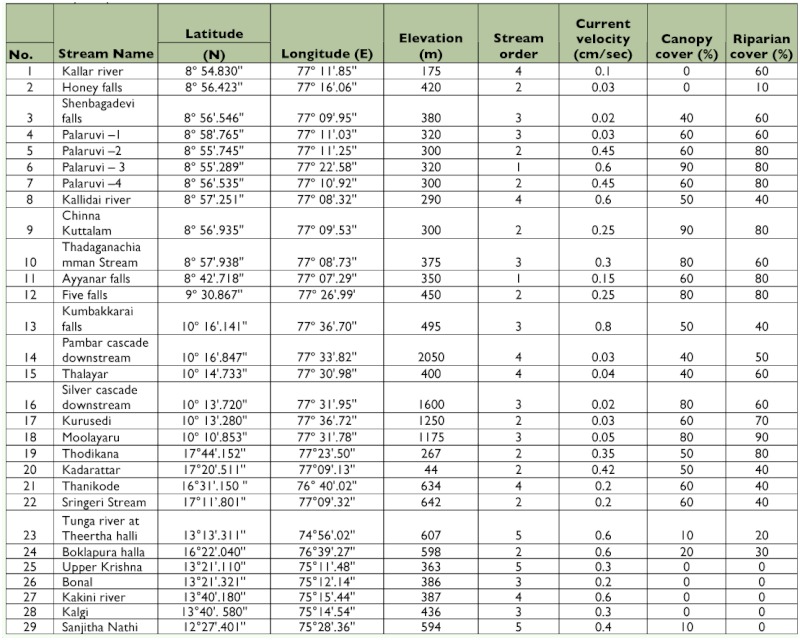
Physical parameters of 29 streams of southern Western Ghats.

## Results

### Physico-chemical characteristic of the study streams

Physical parameters of 29 streams and rivers of southern Western Ghats are given in Table 1. Average temperature among all the sampling sites was 24.8° C. Thalayar had the maximum temperature, and Silver Cascade had the minimum temperature. The mean pH and conductivity were 6.59 and 0.11µ mhos, respectively. Average dissolved oxygen concentrations were 8.62 mg/L. Total dissolved solids levels were low, and mean value was 56 mg/L. Particulate organic matter (leaf litter) ranged from 0% to 50% mg/m^2^. Stream width ranged from 0.25 m to 15 m. The average substrate index was 5.6, being lowest at Kallidai river (2.8) and highest at Palaruvi (7.6) (Table 2). Seasonal variation of the physico-chemical parameters was analyzed in the Thadaganachiamman Stream of Sirumalai Hills between May 2006 and April 2007, and the physico-chemical parameters are given in Table 3. Annual water discharge was high during northwest monsoon season (October and November), and this was gradually reduced and conspicuously absent during summer (May and June).

**Table 2.**
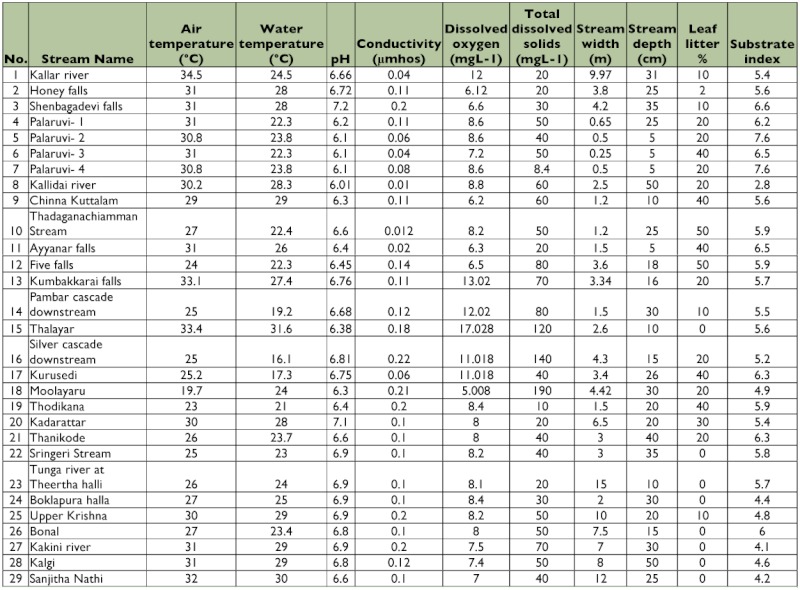
Chemical parameters and stream characteristic features in 29 streams of southern Western Ghats.

### Taxa distribution and diversity analysis

**Figure 2.**
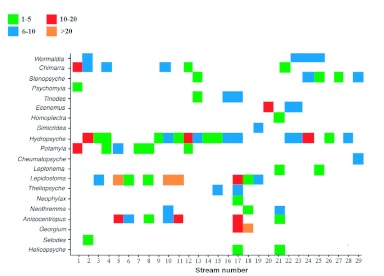
Distribution of taxa (no./m^2^) in each sampling site. Sampling sites are ordered from south to north. High quality figures are available online.

**Figure 3.**
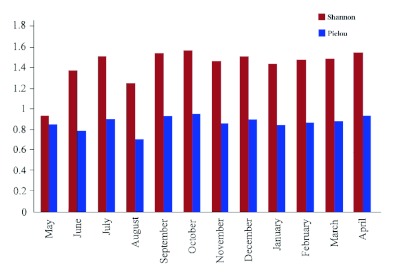
Shannon and Pielou indices for Thadaganachiamman Stream of Sirumalai hills of southern Western Ghats between May 2006 and April 2007. High quality figures are available online.

**Figure 4.**
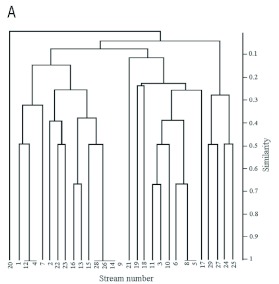
Bray -Curtis dendrogram for caddisfly taxa in 29 streams of southern Western Ghats, High resolution figures are available online.

All totaled, 20 caddisfly taxa were obtained, and the community of taxa differed among sampling sites. Kurusedi had the maximum number of taxa, whereas the Bonal, Kakini River, Kalgi and Sangitha Nadhi of Karnataka harboured only two taxa each. Other sites harbored from 3 to 5 each. *Hydropsyche* had the widest distributional range, whereas *Georgium* and *Helicopsyche* had the narrowest distribution range (Figure 2). Diversity indices showed that the Kurusedi stream had higher diversity and species richness (Table 4). Shannon and Simpson indices indicated that the highest diversity occurred during the month of October in the Thadaganachiamman stream, but the Margalef index showed higher species richness during the month of July (Figure 3). Stream sites 5 to 8; 14, 26, 28, 4, and 12 had the highest faunal similarity than other stream sites. Higher similarity occurred between *Anisocentropus* and *Lepidostoma* (Figure 4).

### Spatial patterns of distribution

**Figure 5.**
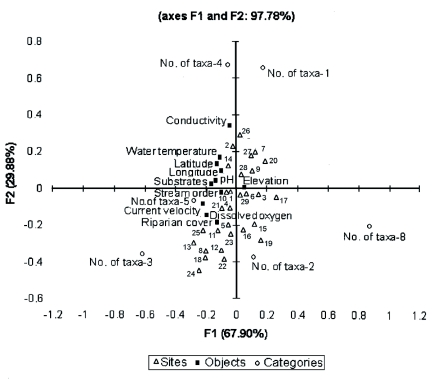
CCA graph representing the relationship between environmental variables and taxa richness in 29 streams of southern Western Ghats, High resolution figures are available online.

Results of the CCA analysis are given in Tables 5 and 6. Eigen values for F1 and F2 axes were 0.007 and 0.003. Cumulative variance was 67.9% for axis F1 and 97.78% for axis F2. Total inertial values were 5.93 (F1) and 2.61 (F2). CCA analysis revealed that among 29 sampling sites, 2 sites represent 4 and 5 taxa each, 12 sites exhibit 3 taxa, 6 sites had 2 taxa, and the seven remaining sites had one. Elevation was an important factor in the F1 axis of CCA. Substrate, pH, and stream order were important in axis F2 (Figure 5).

**Table 3.**
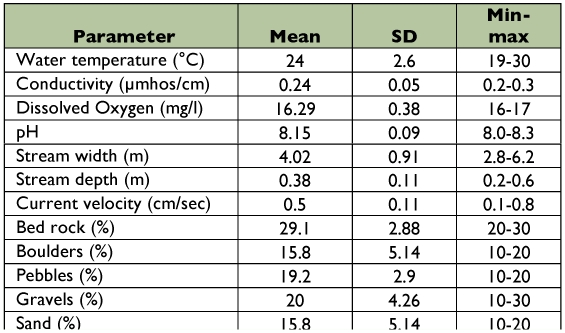
Physico-chemical parameters of Thadaganachiamman Stream in Sirumalai hills of southern Western Ghats.

### Temporal patterns of distribution

**Figure 6.**
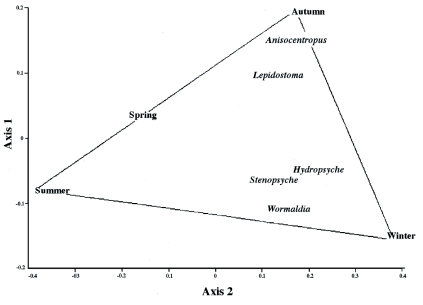
CCA plot representing taxa distribution in the first axis and second axis using seasonality in Thadaganachiamman Stream of Sirumalai hills of southern Western Ghats between May 2006 and April 2007. High resolution figures are available online.

The effect of seasonality on caddisfly distribution was analyzed. Figure 6 shows changes in caddisfly taxa among seasons. Although the first canonical axis explained only 4.2%) of caddisfly variability, the Jolliffe cut-off test indicated that all canonical axes were significantly related with seasonality (0.0199, p < 0.05). *Hydropsyche* and *Stenopsyche* were present frequently in all seasons, whereas *Lepidostoma* and *Anisocentropus* were present only in winter and autumn, and not in summer.

## Discussion

In large scale studies performed in other areas in the world, geomorphological, and other large scale variables such as climate and altitude have been considered as the major factors responsible for macroinvertebrate distribution (Ross 1963; Corkum 1989). Our results are in accord with these findings and suggest that large scale variables were responsible for structuring caddisfly communities. The multivariate analysis suggested that some of the variables (substrate, pH, and stream order) examined in this study influenced the caddisfly distribution and abundance in streams of southern Western Ghats. Apart from these variables, elevation was an important factor.

**Table 4.**
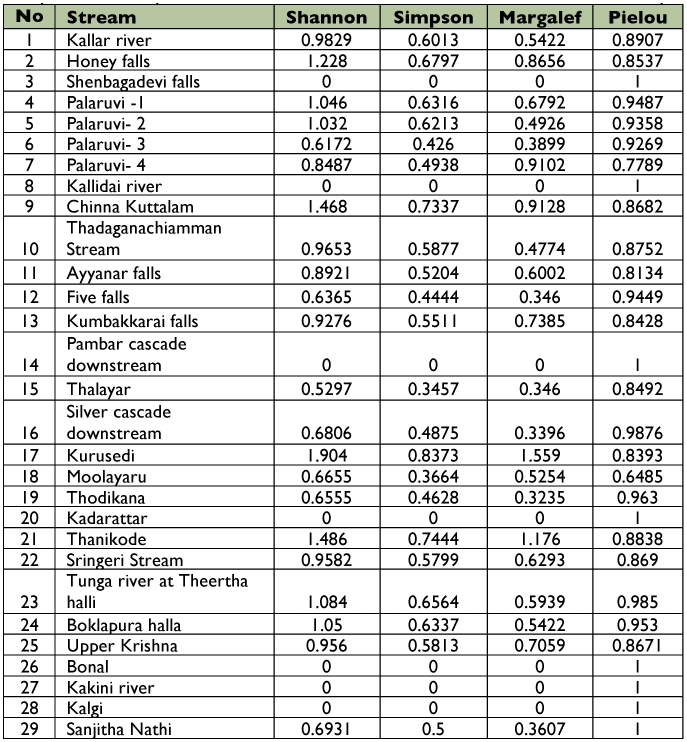
Diversity analysis for caddisfly communities in 29 streami of southern Western Ghats from May 2006 to April 2007.

**Continued.**
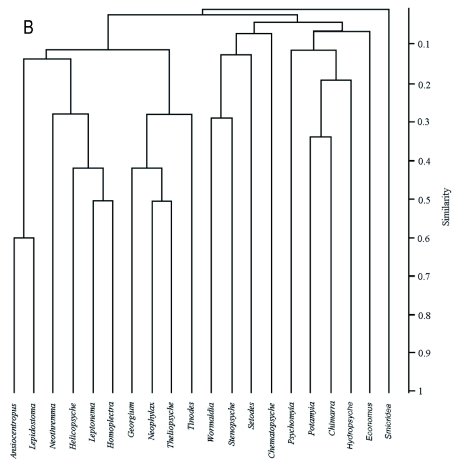

For example, the stream of Kurusedi, which is located at high elevation, had the highest diversity and species richness. The abundance and distribution of lotic invertebrates has been attributed to a variety of factors (Hynes 1970), many of which vary as a function of altitude and thus may be responsible, directly or indirectly, for zonation patterns, Some species were restricted to the headwaters, others to middle or lower reaches, and a few species occurred over wide altitude ranges (Ward 1981), Similarly, *Georgium* and *Helicopsyche* were found at the headwater stream, and *Hydropsyche* was distributed in all stream reaches, This may have been due to flow regime and allochthonous food availability, The present study revealed that seasonality determined the assemblage of caddisfly communities observed during October (northeast monsoon) and July (southwest monsoon), Similar results were observed in the Danubian floodplains in lower Austria (Waringer and Graf 2002), The shredder community of caddisfly (*Lepidostoma* and *Anisocentropus*) was present only in winter and autumn; this may have been due to a substantial amount of leaf litter fall during this season. A similar trend was observed in the lentic water system in south India (Dinakaran et al. 2008).

Physical disturbances and natural environmental gradients were the most important factors regulating the abundance of caddisflies in streams of southern Western Ghats. Physical disturbance in lotic systems was inextricably linked to environmental dynamics and resulting ecosystem and biotic interactions (Allan 2004). The upstream and downstream gradient was an important parameter describing benthic community variation in streams of southern Western Ghats (Dinakaran and Anbalagan 2008). These upstream areas had a higher number of species and acted as colonizing sources for a variety of taxa occurring in the upper study reach that were not found in the lower portions of the study area because of the increased distance from the colonizing sources and anthropogenic impacts. Furthermore, non-point source impacts associated with land use patterns, as well as disturbances by recreational and commercial watercraft, and increases in downstream direction may contribute to environmental disturbances on caddisfly communities. Angradi (1996) observed faunal differences between habitats in Applachian streams. A similar trend was observed for caddisfly communities in streams of southern Western Ghats.

**Table 5.**
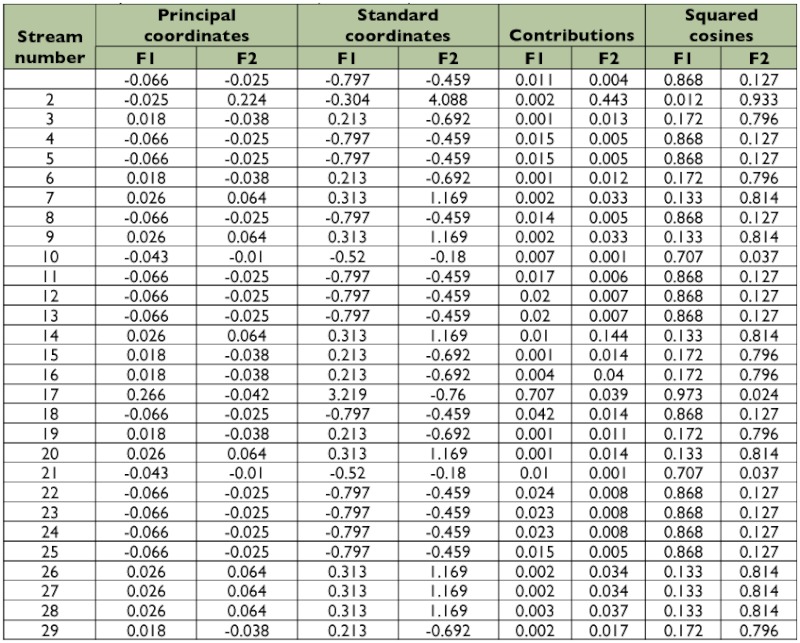
Different non-parametric results of CCA (axis F1 & F2) for 29 streams of southern Western Ghats.

**Table 6.**
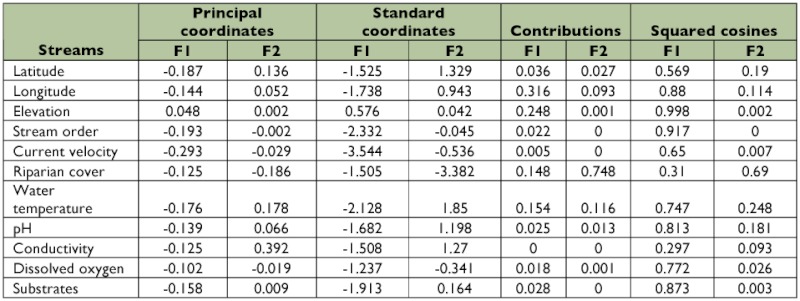
Different non-parametric results of CCA (axis F1 & F2) for environmental variables in 29 streams of southern Western Ghats.

These findings suggest that considerable spatial and temporal dynamics exist in abiotic and caddisfly variability in streams of southern Western Ghats. Multivariate analysis revealed that elevation of the stream played a vital role in the existence and assemblage of caddisfly species. Kurusedi (one of the 29 sampling streams, located among the tourist spots of Palni hills) consisted of rare species of *Helicopsyche* and *Georgium* (not even a single species of *Georgium* was previously recorded in India). Since it lies within a tourist area, this area faces a threat from bathing and washing clothes and vehicles, and it needs to be protected. For example, an ephemeropteran species *Isca* is completely absent due to anthropogenic impacts, but was once recorded in Alagar hills, which is a pilgrimage spot to worship the local deity Theerthakarai (sacred bathing) Raakayee Amman (deity) of Eastern Ghats (Dinakaran and Krishnan 1997). Further studies on the strategies employed with altitudinal distribution and anthropogenic impacts on caddisfly species' ability to persist and maintain their populations in the water body are needed to enhance knowledge of how they survive in unstable and stressed conditions in streams.

## References

[bibr01] Allan J. D. (2004). Landscapes and Riverscapes: The influence of Land Use on Stream Ecosystems.. *Annual Review of Ecology, Evolution and Systematics*.

[bibr02] Anbalagan S. (2005). *Community structure and distribution patterns of aquatic macroinvertebrates in Courtallam hills of southern Western Ghats.*.

[bibr03] Anbalagan S., Kaleeswaran B., Balasubramanian C. (2004). Diversity and Trophic categorization of aquatic insects of Courtallam hills of Western Ghats.. *Entomon*.

[bibr04] Angradi T.R. (1996). Inter-habitat variation in benthic community structure, function and organic matter storage in three Appalachian headwater streams.. *Journal of the North American Benthological Society*.

[bibr05] De Moor F.C. (1992). Factors affecting the distribution of Trichoptera in South Africa.. *Proceedings of the 7th International Symposium on Trichoptera*.

[bibr06] Dinakaran S. (2004). *Biosystematics and biodiversity of Caddisflies in Western Ghats.*.

[bibr07] Dinakaran S, Krishnan N. (1997). Study on diversity of aquatic insects in a tropical stream of Alagar hills, Eastern Ghats.. *Proceedings on Biological Diversity*.

[bibr08] Dudgeon D. (1999). *Tropical Asian streams: Zoobenthos, Ecology and Conservation.*.

[bibr09] Giller P.S., Giller P.S., Myers A.A. (1996). Floods and droughts: the effects of variations in water flow on streams and rivers. *Disturbance and recovery of ecological systems.*.

[bibr10] Jowett I. G., Richardson J., Biggs B. J. F., Hickey C., Quinn J. M. (1991). Microhabitat preferences of benthic invertebrates and the development of generalised *Deleatidium* spp. habitat suitability curves, applied to four New Zealand rivers.. *New Zealand Journal of Marine and Freshwater Research*.

[bibr11] Julka J.M, Vasisht H.S., Bala B. (1999). Distribution of aquatic insects in a small stream in Northwest Himalaya, India.. *Journal of the Bombay Natural History Society*.

[bibr12] Lake P.S. (2000). Distubance, patchiness and diversity in streams.. *Journal of North American Benthological Society*.

[bibr13] Lawton J.H., Hutchings M.J., John E.A., Stewart A.J.A (2000). Concluding remarks: a review of some open questions.. *The ecological consequences of environmental heterogeneity*.

[bibr14] Legendre P., Legendre L. (1998). *Numerical Ecology*.

[bibr15] Leuven R.S.E.W., Vanhemelrijk J.A.M., Van der Velde G. (1987). The distribution of Trichoptera in Dutch soft waters differing in pH.. *Series Entomologica*.

[bibr16] Ludwig J.A., Reynolds T.F. (1988). *Statistical Ecology*.

[bibr17] Magurran A.E. (1988). *Ecological diversity and its measurement.*.

[bibr18] Merritt R.W., Cummins K.W. (1988). *An introduction to the aquatic insects of North America*.

[bibr19] Meyers N., Mittermeier R.A., Mittermeier C.G., Fonseca G.A.B. da, Kent J. (2000). Biodiversity hotspots for conservation priorities.. *Nature*.

[bibr20] Minshall G.W., Robinson C.T. (1998). Macroinvertebrate community structure in relation to measures of lotic habitat heterogeneity.. *Archive fur Hydrobiologie*.

[bibr21] Palmer M.A., Poff N.L. (1997). The influence of environmental heterogeneity on patterns and processes in streams.. *Journal of the North American Benthological Society*.

[bibr22] Poff N.L., Ward J.V. (1990). Physical habitat template of lotic systems: recovery in the context of historical patterns of spatiotemporal heterogeneity.. *Environmental management*.

[bibr23] Poff N.L., Allan J.D., Bain M.B., Karr J.R., Prestegaard K.L., Richter B.D., Sparks R.E., Stromberg J.C. (1997). The natural flow regime: a paradigm for river conservation and restoration.. *BioScience*.

[bibr24] Puckridge J.T., Sheldon F., Walker K.F., Boulton A.J. (1998). Flow variability and the ecology of large rivers.. *Marine and Freshwater Research*.

[bibr25] Ross H.H. (1963). Stream communities and terrestrial biome.. *Archive fur Hydrobiolgie*.

[bibr26] Sivaramakrishnan K. G., Morgan H. J., Vincent R. H. (1996). Biological assessment of the Kaveri river catchment, south India and sing Benthic macroinvertebrates: Applicability of water quality Monitoring approaches developed in other countries.. *International Journal of Ecology and Environmental Sciences*.

[bibr27] Sneath P.H.A., Sokal R.R. (1973). *Numerical taxonomy. The principles and practice of numerical classification.*.

[bibr28] Stewart A.J.a., John E.A., Hutchings M.J., Hutchings M.J., John E.A., Stewart A.J.A. (2000). The world is heterogeneous: ecological consequences of living in patchy environment.. *The ecological consequences of environmental heterogeneity*.

[bibr29] Subramanian K.A., Sivaramakrishnan K.G., Sanjayan K.P., Mahalingam V., Murlairangan M C. (2002). Conservation of odonate fauna in Western Ghats - A biogeographic perspective.. *Vistas of Entomological Research for the New Millennium*.

[bibr30] Subramanian K.A., Sivaramakrishnan K.G. (2005). Habitat and microhabitat distribution of stream insect communities of the Western Ghats.. *Current Science*.

[bibr31] Subramanian K.A., Sivaramakrishnan K.G., Gadgil M. (2005). Impact of riparian land use on stream insects of Kudremukh National Park, Karnataka state, India.. *Journal of Insect Science*.

[bibr32] Suren A. M. (1996). Bryophyte distribution patterns in relation to macro-, meso-, and microscale variables in South Island, New Zealand streams.. *New Zealand Journal of Marine and Freshwater Research*.

[bibr33] Waringer J., Graf W. (2002). Trichoptera communities as a tool for assessing the ecological integrity of Danubian floodplains in Lower Austria. *Proceedings of the 10^th^ International Symposium on Trichoptera*.. *Nova Supplementa Entomologica*.

[bibr34] Wiberg-Larsen P., Brodersen K.P., Birkholm S., Gron P.N., Skriver J. (2000). Species richness and assemblage structure of Trichoptera in Danish streams.. *Freshwater Biology*.

